# Collaborative medication management for older adults after hospital discharge: a qualitative descriptive study

**DOI:** 10.1186/s12912-022-01061-3

**Published:** 2022-10-24

**Authors:** Filipa Pereira, Marion Bieri, Maria del Rio Carral, Maria Manuela Martins, Henk Verloo

**Affiliations:** 1 Institute of Biomedical Sciences Abel Salazar, University of Porto, Porto, Portugal; 2 School of Health Sciences, University of Applied Sciences and Arts Western Switzerland (HES- SO), CH-1950 Sion, Switzerland; 3 Institute of Psychology, Research Center for the Psychology of Health, Aging and Sports Examination, University of Lausanne, Lausanne, Switzerland; 4 Service of Old Age Psychiatry, Lausanne University Hospital, Lausanne, Switzerland

**Keywords:** Older adults, Home-dwelling, Informal caregivers, Polypharmacy, Collaborative medication management, Discharge planning, Qualitative descriptive research, Switzerland

## Abstract

**Background:**

Safe medication management for older adults after hospital discharge requires a well-coordinated, interprofessional, patient-centered approach. This study aimed to describe the perceived needs for collaborative medication management for older adults taking several different medications at home after hospital discharge.

**Methods:**

A qualitative descriptive study was conducted using semi-structured interviews with older adults (n = 28), informal (n = 17), and professional caregivers (n = 13).

**Results:**

Findings revealed four main needs: older adults and informal caregivers’ perceived needs for greater involvement in discharge planning; older adults’ perceived needs to be informed, listened to, and to be actively involved in decision-making; informal caregivers’ perceived needs for help in supporting and coordinating medication management; and older adults’ and informal and professional caregivers’ perceived needs for better communication and coordination between professional caregivers.

**Conclusion:**

This study revealed two underutilized pathways towards improving collaborative medication management: medication follow-up involving a community healthcare professional taking an overarching responsibility and empowering older adults and their informal caregivers in medication management after hospital discharge.

**Supplementary Information:**

The online version contains supplementary material available at 10.1186/s12912-022-01061-3.

## Background

Medication safety is the core theme of the third Global Patient Safety Challenge Programme developed by the World Health Organization [1]. Unsafe medication practices and medication errors are primarily caused by ineffective health systems [1]. Medication errors can occur at many steps in the medication management process, from ordering/prescribing the medication, documenting, transcribing, and dispensing it to when the drug is administered to the patient or during the monitoring of its effects [2]. One of the most common sources of error is poor interprofessional communication and, consequently, poor collaborative medication management [2].

Safe medication management is particularly challenging among older adults due to their increased prevalence of multiple chronic noncommunicable diseases requiring complex multi-drug pharmacotherapy. This is known as polypharmacy when patients take five or more medications daily [3]. Due to age-related changes in pharmacokinetics and pharmacodynamics [4], polypharmacy is particularly problematic in older adults and often leads to potentially inappropriate medications and medication-related harm [5, 6]. Unsafe medication management predisposes patients to adverse health outcomes, which deviate from the original intention of therapeutic benefit and can result in the patient’s physical and cognitive decline and avoidable health costs [7, 8]. Because of its risk for medication-related harm, polypharmacy requires optimal professional communication and collaborative medication management [9]. Effective collaborative medication management occurs when the process of selecting and managing medications results in optimal patient outcomes [10]. It involves an interprofessional patient-centered approach to optimizing medication management and treatment decisions, minimizing medication-related harm, promoting medication adherence, and enhancing medication safety [1, 9].

Collaborative medication management for older adults taking several different medications at home was described by Roughead et al. [11] in their coordinated, integrated, multidisciplinary, patient-centered model of care. This theoretical framework suggested that the medication management of home-dwelling older adults required a patient-centered approach that optimized two-way communication and coordination between professional caregivers and patients, between professional caregivers and informal caregivers, and finally among professional caregivers themselves. This framework also incorporated older adults’ treatment planning and decision-making preferences, despite their different individual profiles, potential willingness to participate, or understanding of their treatment complexity.

Poor collaborative medication management is heightened during care transitions, such as discharge home after hospitalization [1, 8, 12]. Care transitions are a critical period, as revealed by Parekh et al. [13] when exploring older adults’ lived experiences of medication-related harm during the hospital discharge period. Participants felt vulnerable and evoked their reduced capacity to comprehend information, the pressure surrounding the circumstances of their discharge, and a lack of integrated care in the community. In addition, Tomlinson et al. [14] explored older adult patients’ and their informal caregivers’ experiences of post-discharge medication management. Their participants evoked how hospital discharge and changed medication was experienced as a disruption to knowledge, routine, and their ability to manage their medication prescriptions. Various strategies have been used to support medication use, such as creating checklists, seeking advice, or supporting primary care providers to ensure the right medications are supplied on time [14].

Interventions supporting successful care transitions have been developed. The systematic review and meta-analysis by Tomlinson et al. [15] evaluated interventions to support successful transitions of care through enhancing medication continuity. They suggested that interventions bridging the care transition period should continue for at least 90 days after discharge. These were more likely to support successful transitions than those focusing solely on hospital admission or short post-discharge periods. Self-management activities, telephone follow-up, and medication reconciliation were statistically associated with fewer hospital readmissions [15].

Holmqvist et al. [16] explored physicians’ and nurses’ experiences of evaluating older adults’ medications from their perspectives as professional caregivers. According to their findings, working conditions and cooperation with colleagues, older adults, and their professional and informal caregivers, all emerged as important factors related to medication evaluation. Nurses from community healthcare centers (CHCs) described speaking with the older adults and their informal caregivers about continuing treatments and undergoing medication reconciliation so that their medication lists only included medications relevant to the older adult. Communicating with colleagues involved active communication between professional caregivers about how to proceed with an older adult’s medication. This included asking for instructions when information about continuing treatments was missing, and this often involved older adults seeing several healthcare providers [16].

Previous work on safe medication management has relied on clinician-focused or patient-focused perspectives. However, because safe medication management implies collaborative practices using interprofessional, patient-centered approaches, research with a single focus may not have provided a comprehensive description of the phenomenon. The present study therefore relies on a multi-perspective description of older adults’ and their informal and professional caregivers’ needs for collaborative medication management after hospital discharge.

To better explore how home-dwelling older adults with polypharmacy, and their informal and professional caregivers, perceived collaborative medication management after hospital discharge, we triangulated the different perspectives of these three participant groups. The research questions were:

1. What do older adults living at home with polypharmacy and their informal and professional caregivers perceive they needs for effective collaborative medication management after hospital discharge?
2. How is collaborative medication management for older adults implemented after hospital discharge, and what could be improved?

## Methods

### Study design

This study was conducted using a qualitative descriptive design. This naturalistic approach is often used in healthcare research to inform practice by providing a comprehensive, descriptive summary of the perspectives of participants directly experiencing a phenomenon [17, 18]. These different perspectives were captured through individual semi-structured interviews with older adults and professional caregivers and joint interviews with older adults and their informal caregivers and were then analyzed using inductive thematic analysis [19]. Our research team included perspectives from the fields of nursing (FP, MMM, and HV) and health psychology (MB and MdRC).

#### Participants and recruitment

Older adults were recruited in collaboration with nurses from the canton of Valais regional hospital and a CHC, both in French-speaking Switzerland. In Switzerland, CHCs provide support for older adults unable to manage their medications by themselves or with the help of informal caregivers (even if they are autonomous for their activities of daily living). Nurses, usually employed by a municipality, provide CHC services to individuals living in their own homes. Pharmacists are ever more commonly involved in primary care [20].

We used a purposive sampling of older adults returning home with a polypharmacy prescription after hospital discharge. We tried to be as representative as possible with relatively younger and older participants, both sexes, varying periods since discharge (more recent or less recent), and varying numbers of medications. This ensured diversity and a comprehensive summary of their medication management in their own terms [17]. Eligible older adults fulfilling our inclusion criteria (Table 1) were asked for permission to give their names to the researchers. Nonetheless, for ethical reasons, their identities have been coded. There was no previous relationship between the participants and researchers. Based on the systematic review and meta-analysis by Tomlinson et al. [15] suggesting that interventions bridging the care transition should continue for at least 90 days after discharge, FP and MB contacted eligible older adults by telephone between one week and 90 days after their hospital discharge and asked for their consent to participate in the study. To explore a comprehensive, descriptive summary of perspectives about collaborative medication management, older adults without an informal caregiver or support from the CHC were not excluded.

After recruiting an older adult, FP or MB also asked an informal caregiver to participate if the older adult had identified them as the person most involved in their medication management. We defined informal caregivers as any family member, neighbor, or friend assisting a dependent older adult with certain activities in their daily life. That assistance, help, care, or physical presence had to have been given regularly, for at least two of the basic or instrumental activities of daily living (ADL or IADL) or to ensure patient safety, for at least the past six months [21]. Based on our clinical experience, being involved in an older adult’s medication management entails at least one of the following activities: accompaniment to healthcare consultations, assistance in obtaining medication, providing medication, assistance in providing and/or taking medication, monitoring drug effectiveness and side effects, assistance in adhering to medication schedules, and organizing home-care support.

Lastly, FP or MB also recruited a professional caregiver involved in each older adult’s medication management and employed to provide professional community healthcare services. They were invited to participate after an older adult had identified them as the professional most involved in their medication management. The inclusion and exclusion criteria for each group of participants are shown in Table 1. No exclusions were made relative to sex, gender identity, socioeconomic status, or marital status. The enrolment process is schematized in Supplementary File 1.

**Table 1:** Inclusion and Exclusion Criteria for Different Types of Participants

Participants	Inclusion criteria	Exclusion criteria
Older adults	- Aged 65 or above - Hospitalized within the last 90 days - Managing at least five different medications daily	Unable to speak and understand French
Informal caregivers	- Aged 18 or above - Designated by the older adult as the most significant informal caregiver involved in their medication management	Unable to speak and understand French
Professional caregivers	- Designated by the older adult as playing a key role in their medication management	- Student - Apprentice - Unable to speak and understand French

### Data Collection

Data collection in qualitative descriptive research uses minimally-to-moderately structured open-ended individual and/or focus group interviews [17, 22]. Therefore, we used individual semi-structured and joint interviews with older adults and their informal and professional caregivers. We created four interview guides—one for each interview type. Each consecutive interview was based on data collected in the former interviews. The interview guides (Supplementary File 2) were inspired by our literature review and were tested in a preliminary study [23]. Data were collected by two of the research team members (FP and MB).

#### Older adults

Two semi-structured interviews performed two or three weeks apart, each lasting about an hour, were carried out by FP or MB at each older adult’s home. Interviewing participants twice made it possible to analyze any evolution in medication management or collaborative practices following the hospital discharge. However, eight older adults only underwent one interview because the first interview occurred a long time after their hospital discharge date and the data collected in the first interview was sufficiently complete to cover the two planned interviews. We also collected sociodemographic, health, and comorbidity data using the 10th revision of the International Statistical Classification of Diseases and Related Health Problems (ICD-10) [24] and information on prescribed medications taken daily.

The first semi-structured interview collected data on older adults’ perspectives on their transition from hospital to home, the information they had received about their treatment and its possible modifications, and whether their experiences and preferences had been considered in the prescription of their medications.

The second interview essentially focused on medication management at home, the informal and professional caregivers involved in their medication practices, and, finally, how collaborative medication management was implemented after hospital discharge and what could be improved. Older adults were interviewed alone or with their informal caregiver, if necessary. Health restrictions related to the SARS-CoV-2 virus were implemented towards the end of data collection, thus the last two older adult participants were interviewed by telephone.

#### Informal Caregivers

If an informal caregiver designated by the older adult was involved in medication management and had given consent to participate, a joint, third interview with the older adult and their informal caregiver was organized at the older adult’s home one to two weeks after the second interview. Joint interviews provided access to interactions between older adults and their informal caregivers concerning medication management, and to the informal caregiver’s specific tasks in collaborative medication management [25, 26].

The aim of joint interviews was not to triangulate information and arrive at the closest possible ‘picture of reality’, but rather to investigate how each participant’s perspectives intertwined to give a comprehensive summary of their perceptions about collaborative medication management after hospital discharge [27]. Although informal caregivers sometimes expressed themselves more than older adults, this allowed us to better understand their daily interactions and roles in medication management vis-à-vis the older adult and professional caregivers.

Sociodemographic data were also collected. Despite the health restrictions mentioned above, no informal caregivers were interviewed by telephone as the two older adults interviewed this way had not named a significant informal caregiver involved in their medication management.

#### Professional caregivers

When professional caregivers designated by older adults gave their consent to participate, they underwent a semi-structured interview of about one hour. This explored their types of medication management interventions (e.g., prescribing, preparation, administration, monitoring), their frequency, the information received prior to the first visit/consultation after hospitalization (including any changes to usual treatment), how treatment adjustments were being carried out, and the barriers to and facilitators of effective medication management they encountered.

To facilitate the recruitment, and in agreement with the study’s field partners, these interviews took place in professionals’ workplaces (CHC, medical practice, or pharmacy), during working hours, and one to two weeks after the joint, third interview. Sociodemographic and professional data were also collected. Although the primary professional caregivers contacted were interested in participating in the study, their intense workloads made planning appointments for interviews difficult. Five refused to participate and two were unreachable (Supplementary File 1). Therefore, for feasibility reasons, joint interviews involving older adults and their designated healthcare professionals were not planned. Given the health restrictions related to the SARS-CoV-2 virus, the last two older adult participants’ professional caregivers were interviewed by telephone.

### Data analyses

Fifty-three audio-recorded individual and joint interviews were shared among four members of our team (including the two interviewers) and fully transcribed. The analysis was conducted in French, the data’s original language, although the quotes used to illustrate our findings have been translated into English. Each full transcript underwent an inductive thematic analysis by the same four team members to identify common patterns in the full dataset [19, 28]. An inductive approach means that the themes identified were data-driven (rather than theory-driven) and that during the coding process the researchers did not try to fit the data into a pre-existing coding framework [19]. Individual and joint interviews were considered equally important to capture different perspectives of the phenomenon. Data not focused on an older adult’s medication management and their most recent hospitalization were not considered useful for answering our research questions and were not coded. Although we did not conduct a separate analysis of each specific healthcare profession involved, we have distinguished their particular perspectives in the results. Moreover, we noted caregivers’ years of professional experience but did not include them in our data analysis due to the limited number of professionals participating in the study. The four researchers met regularly to check whether patterns matched the coded extracts across the entire data set and until a consensus was reached. This analysis procedure enabled a systematic classification of all the descriptions into distinct themes, framed by the similarities and differences in participants’ accounts. The entire corpus of interviews was then reviewed to validate the themes that had emerged. The analytical process was completed when *patterns of shared meaning* were reached regarding participants’ accounts and our research questions [28]. Preliminary results were then discussed with the other authors. This widely acknowledged method of qualitative descriptive research fitted well with the study’s aims as the topic emerged from a clinical concern and the participants were those directly affected by the condition [22].

### Trustworthiness

The four key components of trustworthiness identified by Lincoln and Guba [29]*—*credibility, dependability, confirmability, and transferability*—*can readily be applied to qualitative descriptive research [22]. In the present study, credibility was ensured by reviewing the interview data collected by MB and FP, by each interviewer writing notes on the interview process to ensure that each aspect followed the interview guidelines, and by conducting a briefing before each interview. Confirmability was ensured by the team members evaluating the research process during meetings and reading and analyzing the data together, by describing participants’ demographic data, and by including their direct quotations. Dependability was ensured by defining clear study stages, keeping research diaries, having regular weekly coordination meetings, and ensuring accurate data coding. Strategies to support transferability included purposeful sampling according to our inclusion and exclusion criteria and providing a description of participants and the context of their perceptions.

## Results

After completing 28 interviews with older adults, no new information emerged, suggesting that data saturation had been reached [22]. Consequently, no further participants were included. The sociodemographic characteristics of the three types of participants are presented in Table 2. The median older adult had 12 ICD-10 diagnoses when in hospital (range 3–27) and eight prescribed medications at hospital discharge (range 5–21). Only 17 informal caregivers participated in the study because not every older adult had an informal caregiver, not every informal caregiver was involved in medication management, and not all of them consented to participate. Thirteen of the 17 informal caregivers interviewed lived with the older adult, three lived in the same town or village, and one lived outside the canton. Most (n = 11) provided daily assistance in multiple activities (IADL or ADL). Older adults’ levels of autonomy in medication management were very heterogeneous. Whereas some prepared and took their medications independently, others needed their informal caregivers and/or health professionals (a nurse or pharmacist) to prepare and administer them. The types of medication management assistance provided were also variable and depended on coordination between the different stakeholders involved. Older adults designated three types of healthcare professionals involved in their medication management: nurses, pharmacists, and general practitioners (GPs). More information on participants’ characteristics is provided in Supplementary Files 3 and 4.

**Table 2:** Participants’ Sociodemographic and Professional Characteristics and Older Adults’ Polypharmacy and Medical Status (ICD-10 Diagnoses)

Sociodemographic and professional characteristics	Older adults (n = 28)	Informal caregivers (n = 17)	Professional caregivers (n = 13)
Sex (number)			
Female	11	15	10
Male	17	2	3
Age (years)			
Median	83 [66–94]	67 [48–86]	45 [28–58]
Relationship with the older adult			
Spouse/partner		10	
Child		6	
Daughter-in-law		1	
Profession (number)			
Retired	28	9	
Employed	0	7	
Unemployed	0	1	
Nurse			5
Pharmacist/Pharmacy Assistant			4
General Practitioner/Specialist			4
Number of drugs			
Median	8 [5–21]		
Number of ICD-10 diagnoses			
Median	12 [3–27]		

Our qualitative descriptive analysis revealed four main themes about collaborative medication management:

- older adults’ and informal caregivers’ perceived needs for greater involvement in discharge planning;
- older adults’ perceived needs to be informed, to be listened to, and to be actively involved in decision-making;
- informal caregivers’ perceived needs for help in supporting and coordinating medication management;
- older adults’ and informal and professional caregivers’ perceived needs for better communication and coordination between professional caregivers.

### Older adults’ and informal caregivers’ perceived needs for greater involvement in discharge planning

All older adults and informal caregivers perceived hospital discharge as a desirable but distressing moment. Discharge planning was organized by physicians, ward and liaison nurses, and involved setting up or reactivating the medication management services (deliveries, weekly pill-box preparation, and administration) provided by CHCs. However, the older adults and informal caregivers interviewed did not always appreciate how discharge home was organized by the hospital’s healthcare professionals. Indeed, some refused medication management by their CHC after hospital discharge despite the recommendations and prescriptions written by hospital professionals.

Other participants felt that they had been insufficiently listened to and consulted when decisions were made during hospital discharge. They described episodes of poor communication between hospital departments and few opportunities to discuss things with hospital healthcare staff. For example, OA20, living alone and hospitalized following a pulmonary embolism, complained about the lack of explanations he received at the hospital when the medication that he had been taking for many years was changed.

They could have talked to me about it before stopping it [Zoldorm®]. (…) They’re all assistants, lots of them… There’s some big professor on each floor, the rest are all assistants with their computers on wheeled stands and they type as they walk along. That’s it, eh. They don’t say much (…). They don’t communicate with anyone. They talk among themselves. They come in together; they leave together.

Other older adults described having experienced the process of their hospital discharge as being too rapid or even happening too early. This was described by OA01’s two informal caregivers. They were surprised by the decision to discharge their mother, who lived alone, to her home when she presented with significant functional impairments. Furthermore, the speed with which hospital healthcare professionals made that difficult decision left no time for the informal caregivers to discuss it or ask questions about changes to their mother’s medication.

IC01b: “Honestly, I found it all a bit strange because… with, umm, her doctor, she said that at a certain age, you shouldn’t unsettle them too much. When they are used to a certain medicine, you shouldn’t make too many changes, because you often, you know? And then, suddenly, I said to myself, ‘Why are there all these changes when she’s used to certain things.’ ”.

Interviewer: “And you never had the opportunity to talk about it?”

IC01b: “No. No, because in fact… at the same time, we didn’t expect her [OA01] to be out so soon. And so, all of a sudden, huh, it was all done rapidly.”

Some informal caregivers expressed their fears about older adults’ discharges home occurring too early. They developed strategies to mediate this and prevent potential health complications, including reorganizing their families to ensure a more frequent or regular presence with their older adult relative, especially regarding mealtimes and medication schedules. As in OA18’s case, hospitalized following a stroke, an intermediary stay in a rehabilitation clinic was sought. His wife (IC18) did not actively assist him with his medication management, but they seemed to provide each other with mutual support and took their medication at the same time in the same place. According to IC18, her husband was discharged from hospital too soon and should have been prescribed an intermediary stay in a rehabilitation clinic.

In summary, this theme described older adults’ and informal caregivers’ dissatisfactions with discharge planning after hospitalization. Several older adults and informal caregivers described not feeling sufficiently involved in the decisions concerning their transition from hospital to home.

### Older adults’ perceived needs to be informed, to be listened to, and to be actively involved in decision-making

Many older adults described their needs to be listened to and to be involved in decisions concerning their medication management after hospital discharge. This theme pointed to two extreme attitudes along a continuum. At one extreme, we found older adults who wished to control their own medication, and at the other, we found some who described themselves as having lost their ability to manage it. Older adults with the former profile strived to maintain control over their medication, often able to remember what they had already taken and what they had to take next, even if they could not always name all their prescribed medications. They felt it was crucial to maintain autonomy in their medication management, and any loss in independence would be difficult to accept.

OA22 explained her need to be informed about her medication during her last hospitalization, following pneumonia, and she compared herself to other patients.

*“(…) I said to my son, ‘Bring everything to the hospital.’ And I even had him bring the box that was in the refrigerator. I said they had to put it in the fridge there, because I don’t want to be changing my medication 36,000 times. Maybe I’m a pain, but that’s the way it is. You see, I want to manage things depending on whether they’re any use. I don’t want to take anything just for the sake of it. You understand? (…) So, there are some people who don’t worry about that. They… they swallow their pills any old way. No, me, I want to know what I’m taking. And why. (…) So, I don’t know why they change my medication without telling me. They just give you the prescription, and that’s it. No, me, I want to know the whys and wherefores.”*.

Nevertheless, most of the participants only realized that their medication had changed, or that new medication had been added while they were in hospital, once they had got home. This was the case with OA05, hospitalized following a diagnosis of rectorrhagia. She received one new medication in hospital, but only learned about its contraindications on her return home; she decided that one of them matched her profile and decided to stop taking it without consulting her GP.

She’d given me some sleeping pills [Distraneurin®]. Then I had a look. And it was written that if you had a cough, and all that, then you shouldn’t take it. So, me, because I had asthma and I was coughing, I didn’t dare take it.

In addition, OA22 explained her efforts to maintain control of her medication preparation at home and how this was not always well understood by professional caregivers—in this case, by an independent home-care nurse caring for her after her discharge.

(…) I’d laid it all out here, and I said to her, ‘You can ask me which medication is which.’ Then she [the independent home-care nurse] said to me, ‘Give it all to me.’ I said, ‘No. I won’t let you put your hands in my medication drawer because they’re…’ You see, they are always set out in the same way: Lisinopril®, Plavix®, Atorvastatin®, Trajenta®. Everything’s set. I don’t want anyone sticking their hands in there and moving it about. So, I said, ‘You ask me, and I’ll give it to you.’

Older adults who wished to control their own medication sometimes sought to prove, from the outset, that their cognitive faculties, such as their decision-making capacities, were intact. OA10*—*hospitalized for lower back pain*—*underlined his need to control his medication management, which he did by deliberately not adhering to some medications given to him in hospital and after discharge home.

*“No, because even at the hospital, I refused to take any medication, eh! (…) I want to have some control. (…) Yesterday, nothing. Today, nothing [Dafalgan®]. (…) I took that decision. Why? I’ve already got a patch. It’s the patch I’ve got against pain. If I’ve then got to take the Dafalgan® too, then I’ll intensify these analgesics. What’s the good of that? If it put me in a state of… No! (…) No, I’m not bedridden, me. Even if I was in that state, I wouldn’t take that. (…) she [the CHC nurse] was supposed to change the patch for me, and I said that I don’t want it. We’ll see how it’s going on Friday*.”.

At the other extreme of the continuum, some older adults talked about being resigned to their health status, accepting decisions made about their medications, and obeying the professional recommendations made to them. For example, IC15 underlined her husband’s continued acceptance and passivity.

“*(…) He’s a very agreeable patient, because he’ll never contradict, never… He lets himself be cared for. I think that’s also part of the reason that he’s still here. (…) He’s brave, but he’s passive too. That’s to say, he may get annoyed inside, but he doesn’t show it. I can’t do it, but he listens to what he’s told and then he does what he’s told. He accepts it—that’s why he’s still here.”.*

Resignation seemed to be built on trust in professional caregivers, as described by OA01 with regards to her medication management.

They did what they were supposed to, huh. I couldn’t say, ‘No, I don’t want those particular pills.’ Why? Because I was in their hands. I had to do what they wanted, you know?

To different degrees, these older adults all let go of any active participation in their medication management and they handed over the responsibility to their informal and professional caregivers. Informal caregivers noticed these changes in attitude, as IC01a (daughter of OA01) described.

(…) she’s come to a stage where she doesn’t give a damn. (…) She’s arrived at a stage in her life where she tells herself, ‘Well, I don’t have any choice, do I?’

Other participants with this sort of profile had a rather obedient attitude but nevertheless retained an interest in their medication. One example was OA18, who very frequently solicited his pharmacist (Prof18) to ask him questions about his treatment, probably to progressively regain control in the same rigorous way he took his medication.

To summarize, despite some extreme attitudes along a continuum, most older adults described, at different levels, their desire to be informed, to be listened to, and to be involved in decision-making about their medication management at home after hospital discharge.

### Informal caregivers’ perceived needs for help in supporting and coordinating medication management

Informal caregivers described the diverse tasks they carried out to ensure optimal medication management and their older adult’s safety following discharge home. They also described the negotiations they took part in regarding medication management coordination. These elements ensured effective medication management and the older adult’s safety, helping to prevent possible adverse health outcomes. Some informal caregivers, like IC12, equated institutionalization to a failure of the coordination system in place.

Me, I say, he’s lucky, because if we weren’t here, he’d be in a care home.

Another informal caregiver (IC11) went to the hospital to collect a prescription for Tramal® on realizing that their older adult relative had not been discharged with it. On discharge home, other informal caregivers (IC25, IC04, IC17b) went to pick up medication at the pharmacy, prepared pill-boxes, or drew a visually pleasing medication schedule. They also administered medication, accompanied older adults to medical consultations, and participated in ADL. This was also emphasized by older adults, as when OA01 explained that because she “*can’t do anything anymore*”, her two daughters had to do lots of things for her instead.

Numerous descriptions of negotiations with professional caregivers revealed the central role informal caregivers played in older adults’ day-to-day medication management. The informal caregivers interviewed tended to support the wishes of their older adult relatives, often playing an active role in sharing out tasks across the older adult’s care network, in addition to completing tasks themselves. Although informal caregivers took on some functions, they described needing professional help with others. OA01’s two daughters took care of remembering and administering medication, but they asked the pharmacy to prepare the pill-box in advance and the CHC for daily monitoring and support in the ADL and IADL. Furthermore, some informal caregivers set limits to their roles.IC15, for example, decided to stop managing her husband’s medication and ask for professional help.

So then I said, ‘I’m not taking care of that. I’ve got other things to do, and that’s too delicate’, and then because I’m anti-medication, I don’t want to touch it.

Some informal caregivers, like IC02, IC04, and IC07, spontaneously spoke about how they were overloaded and of the burden of ensuring that care transitions and home-care services functioned properly. As IC04 described:

I’m exhausted from having to coordinate with all the different professions who don’t do things the same way—with all those people, and it’s not my field, etc. And then, at the same time, you have to try and make a good impression. Give them a big smile. ‘How are you? Is everything all right?’ etc. And then continue making meals, etc.

To summarize, this theme highlighted the complexity of the support informal caregivers provide to medication management and their needs for help to keep supporting their older adult relatives and coordinating the different professional caregivers involved. Informal caregivers’ expectations of the role of professional caregivers in medication management after discharge from hospital did not always correspond to the care actually provided.

### Older adults’ and informal and professional caregivers’ perceived needs for better communication and coordination between professional caregivers

According to the professional caregivers interviewed, an effective medication management transition from hospital to home began before hospital discharge and was dependent on communication between the health and social care professionals involved in discharge planning. This involved exchanges between the liaison nurse and home-care services about the treatments prescribed at hospital discharge. After discharge home, several professional caregivers were involved in medication management: GPs, home-care nurses (from the CHC, free-lance, or in protected accommodation), and sometimes, depending on the older adult’s needs, pharmacists, physiotherapists, CHC occupational therapists, dieticians, and specialist physicians.

Older adults placed GPs and home-care nurses at the center of their support networks for medication management and assigned them the role of coordinating and collecting all the relevant information, especially information coming from the hospital. Because they knew the older adult before their hospitalization and were easily available to respond to problems or questions, they could verify whether new medication interfered with or affected an old one and whether any medication should be suspended, stopped, or replaced. Several participants cited home-care nurses as the professional caregivers most involved in their medication management, assuming a coordination role. We noted that this was often the case among the more functionally impaired older adults, whether living alone or with an aged partner, such as OA15, who was hospitalized following a stroke. His wife, IC15, was central to his health management, as evidenced in their two interviews, in which her husband participated only minimally. Given OA15’s level of dependence, IC15 asked for assistance from the CHC following her husband’s hospitalization, notably to prepare his weekly pill-box.

(…), but we have to have a nurse come around. I don’t want to take care of the medication anymore. They [the CHC nurses] prepare the weekly pill-box (…) They order the medication; they do everything. (…) Oh, yes, and its [the nurse] who checks it. When anything’s missing, it’s her who makes the order for new medication. (…)

Collaboration between GPs or CHC nurses and community pharmacists were only occasionally mentioned. Prof01 was a pharmacist designated by OA01’s two daughters as the healthcare professional who contributed most to their mother’s day-to-day medication management. Prof01 explained that the hospital or the liaison nurse faxed or sent a secure email in advance so that the community pharmacy could order and prepare particular medications that might not be in stock; in cases of doubt, they contacted OA01’s GP.

IC12 was the daughter of OA12, hospitalized following decompensated chronic obstructive pulmonary disease. She explained the reasons why she solicited the help of a pharmacy for the preparation of OA12’s weekly pill-box.

*(…) because before we did it ourselves as there were not too many quarter- or half-pills. And then it changed. One day it’s so many pills and so many half-pills and quarter-pills, and after that, the next day, it changes. It’s so we don’t get it wrong. We wouldn’t want to get it wrong.*

IC12 also described the complex day-to-day coordination between the professional caregivers involved in OA12’s medication management.

So, it’s the pharmacy [that prepares the medications]; we have the sheet. Every time we leave the hospital, they provide us with the sheet with which medications to take. And then afterwards, there’s the nurse who comes once a week to give the Sintrom® as well. To check on the Sintrom®—and on us, really—the nurses come twice a week to check his weight, blood pressure, and report to the doctors. (…) She [the GP] does home visits if it’s really necessary.

According to Prof01 (a pharmacist) and Prof03 (a CHC nurse), the collaboration between pharmacists and home-care nurses was recently strengthened thanks to a regional pilot project on the prevention of adverse drug events. As Prof03 explained, this offered patients with polypharmacy the chance to have their weekly pill-boxes prepared in a pharmacy instead of in the older adult’s home by a home-care nurse, with the aim of promoting medication reviews and double-checking by professionals:

*“Yeah, in fact, we put that in place so that we could delegate the preparation of weekly pill-boxes to pharmacies. So, when the pill-boxes are simple and they don’t change too much—because the situations at home aren’t too complex—then we ask the pharmacies to prepare the pill-boxes and deliver them to the CHC. And we take them to people if they can’t collect their pill-boxes. We deliver them directly to their homes. But we’ve got fewer and fewer weekly preparations to do; we only keep the complex weekly pill-boxes—those with frequent changes and unstable situations.”*.

Although collaboration during care transitions seemed to work well for some participants, others raised points of concern. From the professional perspective, one GP (Prof21) and one home-care nurse (Prof04) considered it potentially harmful that there was often a long delay between when they were called upon to intervene in care transitions after a hospitalization and the moment when they received the paperwork informing them of discharges. This was particularly relevant when there were changes in medication treatments, as for OA04. At her discharge, following hospitalization for a fall at home, OA04 and Prof04 recalled that there was a lack of coordination. Prof04, a nurse in the protected accommodation where OA04 lived, described the difficulties experienced when the hospital failed to transmit new prescription and discharge documents to the GP. After OA04 had an adverse reaction to her medication, she explained:

(…) I called the doctor and he said to me, ‘But I haven’t received any paperwork.’ There was no information on her… (…) I find that there is often a lack of communication, or sometimes the hospital tells us that they are sending us the discharge documents, but we don’t receive anything.

Indeed, communication by post sometimes seemed less effective than wished for, as one GP (Prof21) confirmed. He wanted discharge letters or consultation notes to be transmitted earlier because delays could lead to misunderstandings and errors.

Poor communication could also occur between professional community caregivers like home-care nurses and GPs, compromising the quality of medication management and perhaps even the older adult’s safety. This was described by OA28, living alone and hospitalized for abdominal adhesions with obstruction.

(…) I said to [the CHC staff], ‘But if I get something like I got last time, what good does it do if the information doesn’t go any further? You should inform my GP. That’s the least you could do.’ I always thought that those sorts of reports got made. (…) So, I said, ‘Listen, now. At all costs, when something special occurs, like last time, you come by on Tuesday morning, and in the afternoon, I should go to the emergency department. Have you noted that down? You’re writing lots down, but…’ (…) So, I said that it wasn’t normal that my doctor wasn’t informed. I had to inform her myself. Yes!

IC04 also described her dissatisfaction with the CHC staff’s communication and coordination and the need for more effective medication management of her mother’s pain.

*“(…) And, in fact, when we looked, we noticed that quite a few errors had been made by people all along the chain of care. (…) We’ve got a table where we note which day we have to put which patch on [Fentanyl®]. Every 72 hours, they’ve got to be changed. (…) There was this mismatch where, in fact, she found herself changing the patch every 24 hours, and then later, there was no patch for 4 days until I called up the CHC. The person in charge said to me, ‘But Madame, you just asked for assistance, not for a…’(…) They knew we had to put on a patch, but she couldn’t authorize it. She didn’t do it. I said to her, ‘So you can see a person suffering right in front of you, you know that they need to put on a patch, and you don’t do it. Do you find that normal?’ ‘Well, yes. You asked for a home-help, not a home-nurse, etc.’ So, it’s been an ordeal getting this accepted, etc., getting them to accept it.”*

These organizational difficulties, just for changing a Fentanyl® patch, were reiterated by Prof04, the nurse working in the protected accommodation where OA04 lived.

(…) So, there were often problems like that. I think that because there are several of us… (…) It’s true that collaboration is so important, that information gets transmitted from the hospital to the CHC, to doctors and to us. Even if we are not a medical care institution, it’s still important that we get the documents.

In summary, several actors—both professional and informal—were involved in different ways depending on the complexity of the older adult’s clinical picture and the informal care resources at their disposal. However, all the interviews pointed to a perpetual search for better communication and coordination between professional caregivers for more effective collaborative medication management.

## Discussion

Our findings provided a description of older adults’ and informal and professional caregivers’ needs with regards to collaborative medication management after hospital discharge. Four main needs emerged from our analysis. Whereas some of those needs were common to all the actors involved, others were specific to just one group.

*Both older adults and their informal caregivers expressed a need for more involvement in discharge planning.* Not only was discharge planning often considered unsatisfactory or absent, and discharge home inadequately organized, but older adults also felt insufficiently listened to or involved in discharge planning, corroborating recent findings in Switzerland [30]. Following their discharge from hospital, older adults and their informal caregivers perceived medication management at home to be complex during this period of instability, where existing strategies to support care coordination came under pressure. From our interviews, there was no *usual* procedure for ensuring optimal medication management for older adults after hospital discharge (e.g., being called in for a medication review by a GP). Older adults and their informal caregivers were confronted with more functional impairments during these transition periods and the uncertainty of whether they would be able to manage their medication and remain in their homes in the future. This was in line with Parekh et al. [13] findings about older adults’ lived experiences of medication-related harm during the hospital discharge period. These revealed that care transitions were a critical period, when older adults felt vulnerable and less confident about understanding information. Tomlinson et al. [14] found that older adult patients and their informal caregivers evoked how hospital discharge and changed medication was experienced as a disruption to knowledge, routines, and capability. Even if older adults’ and informal caregivers’ perceived needs for greater involvement in discharge planning have been revealed in previous studies, our findings emphasized that patients were still not sufficiently involved, despite discharge planning’s importance for successful care transitions [1, 8, 12]. This may be explained, although not justified, by the current worldwide trend towards shorter lengths of stay, which places care transitions under increased pressure [31]. In Switzerland—where the healthcare system combines public and private funding—the cantons cover 55% of inpatient treatment costs and health insurance companies cover 45%, whereas for outpatient care, the health insurance companies cover 100% of costs. Cantonal authorities, therefore, are trying to shorten hospital lengths of stay as much as possible and favor outpatient treatment [32], although this overstretches community services.

Our findings also underlined that unsafe medication management was an important issue in care transitions. This was in line with the results of Lee et al. [33], who identified medication as one of the five principal themes influencing care transitions. Moreover, there have been recent advances in the development and application of innovative care pathways and multidimensional discharge planning [34, 35]. Nevertheless, inadequate patient assessment in acute care settings—leading to a lack of knowledge about patients’ social circumstances—followed by poor communication between hospitals and community care providers remains a cause of discontinued, fractionated care [34, 35]. Hospital to home discharge planning by a collaborative interprofessional team, including service referral and education for patients and informal caregivers, has been shown to be effective at providing safe care transitions for older adults with multiple chronic conditions [15]. The advantage of an interprofessional approach is linked to different professionals’ abilities to identify and meet the older adult’s medication management needs.

*Older adults described their needs to be informed, to be listened to, and to be involved in decision-making*. While some older adults strived to retain, regain, or consolidate control over their medication management and decisions (sometimes leading to confrontations with professional caregivers), others were resigned and adopted recommendations passively. The need to be proactively involved in their medication management may represent an underexplored resource in the pursuit of medication safety among older adults living at home with polypharmacy. For some participants, maintaining control over their own medication management was a means of displaying their autonomy and cognitive capacities, reinforcing their ability to live at home and thus removing the risk of institutionalization in a nursing home. Still being able to manage one’s medication was, therefore, a way for some older adults to legitimize them remaining at home. Holmqvist et al. [36] described similar findings in their study about home-dwelling older adults’ experiences of evaluations of their medication treatments. Their participants revealed that they were willing to be actively involved in their medication evaluations. Even though older adults trust that their physicians will regularly undertake these evaluations, that is not necessarily the case [36], which contributes to an increased risk of medication-related harm. This is in line with Roughead et al.’s coordinated, integrated, multidisciplinary, patient-centered model of care, which states that patient safety can benefit from a co-production approach to medication management, with professional caregivers explicitly sharing information with older adults and agreeing on responsibilities related to ongoing medication treatment [11]. Our findings add knowledge by revealing two types of obstacles to older adults’ involvement in their medication management: those of an individual nature, such as a loss of autonomy or cognitive faculties, and those to do with collaborative practices, such as difficulties communicating within and across the care network or the absence of a healthcare professional with an overarching responsibility for the patient. Professional caregivers should consider the obstacles to older adults being involved in their medication management, including the patient’s personal preferences and values regarding discharge planning [30].

*Informal caregivers described their needs for help in supporting and coordinating medication management*, including complex negotiations to optimize care coordination. They also emphasized their work overload and the burden of ensuring that the healthcare network functioned properly to ensure safe medication management. The present study showed that informal caregivers played a vital role in ensuring safe and appropriate medication use by home-dwelling older adults, especially those who may also have a cognitive impairment. Some participants, however, reported complications related to this activity, including the time spent, anxiety about making mistakes, and solving communication problems or disagreements with professional caregivers regarding medication practices and care coordination. Previous studies showed that informal caregivers could also face uncooperative behavior from their older adult relative, difficulties maintaining continuous supplies of medication, problems administering drugs, and the need to make clinical judgements (e.g., in response to side effects or about over-the-counter medication) [37, 38]. Whereas some informal caregivers in our study expressed their worries about their older adult relative’s health and the day-to-day efforts made to ensure effective care transitions and medication management, older adults themselves rarely brought up their relational and affective links with their informal caregivers. Although this finding went beyond our research questions, other studies have revealed that the frequency with which gratitude was expressed was significantly associated with caregiver burden [39] and that greater perceived gratitude was positively associated with informal caregivers’ psychological well-being [40]. Interventions by professional caregivers to improve informal caregivers’ mental health might not only reduce the burdens of their role but also enhance their perceived gratitude and support their involvement in medication management.

*Older adults, informal caregivers and healthcare professionals all expressed the perceived needs for better communication and coordination between professional caregivers*. They described how numerous healthcare actors were involved in medication management after hospital discharge, with GPs and home-care nurses being at the center of the healthcare team. Home-care nurses were more involved in medication management with the most dependent older adults. They also described how dysfunctional communication within healthcare teams compromised the quality and safety of medication management. Medication management was put at risk by the long delays before professional community caregivers received notifications about hospital discharge, particularly if there were changes in medication treatments. Our findings also revealed, perhaps unsurprisingly, that the more actors were involved, the more complex interactions became and the more difficult they were to coordinate, which is in line with Cramm et al. [41] and Bindels et al. [42]. There were diverging points of view, for example, on when and why the preparation of weekly pill-boxes should be delegated to community pharmacies. Whereas pharmacists considered that they only dealt with more complex treatments, two nurses interviewed mentioned that pharmacies took care of “simple” treatments. The notion of what constitutes complexity clearly seemed to differ between pharmacists and nurses. Pharmacists seemed to focus on the complexity of the medication regimen, whereas nurses looked at the complexity of the medication management that would have to take place in older adults’ homes (including functional limitations, values and preferences, and the availability of informal caregivers).

As argued by Roughead et al. [11], communication between the different actors involved in older adults’ care—both professional and informal—was considered a key element of well-functioning medication management, particularly during care transitions. However, our findings showed that, in certain situations, communication was clearly lacking, putting effective medication management at risk and, consequently, the quality and safety of older adults’ care. Collaborative practices for recently discharged older adults should entail safe medication management and attention to communication, education, competency development, clinical support systems, and policy and regulatory decisions [10, 15]. Interventions that foster communication and collaboration between multiple stakeholders, particularly during care transitions between hospital and home, have been shown to reduce the worsening of chronic conditions and the significant costs of hospital readmission [43]. These interventions include bundled, multi-stakeholder approaches to best meet patients’ needs.

**Fig. 1:**
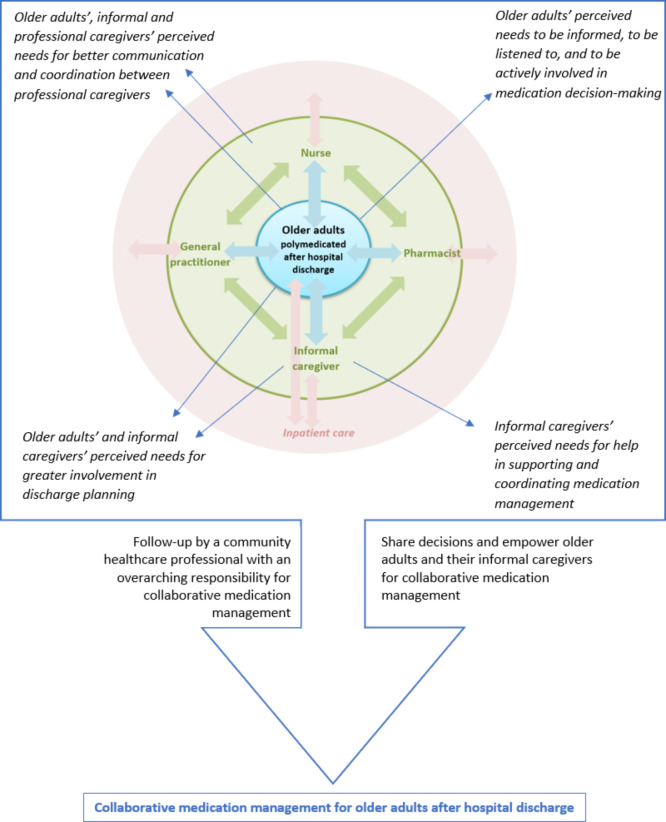
Overlap between older adults’ and informal and professional caregivers’ perceived needs for collaborative medication management after hospital discharge

Our findings also suggested that once an older adult has returned home with a polypharmacy prescription, follow-up by a community healthcare worker skilled in care management and with established community links to various other services could be a more efficient way to manage the complex medication required for multiple chronic conditions [34, 44]. This role could be performed by a primary care nurse manager, as our findings emphasized that nurses were the professional caregivers who interacted most with all the actors involved in medication management. Nurses’ essential role in providing collaborative medication management, given their direct patient contact and their collaborative relationship with physicians, has been documented previously [10]. In addition, our findings revealed older adults’ perceived needs to participate in medication decision-making, which requires empowering patients and their informal caregivers [43, 45–47]. By facilitating older adults’ empowerment, healthcare providers promote collaborative medication management and shared-decision making, which could encourage them to take control of their own health, with the endpoints of safe medication management and better patient outcomes [48]. Older adults could benefit from a joint empowerment and monitoring approach, with a community healthcare professional taking on the essential overall responsibility for sharing information and tasks related to medication management (see Fig. 1).

### Study strengths and limitations

To the best of our knowledge, scarce qualitative descriptive studies have analyzed the needs for effective, collaborative medication management after hospital discharge expressed by older adults taking several different medications at home and their informal and professional caregivers. According to this multi-perspective description, safe medication management requires collaborative practices aimed at patient-centered care. The present paper adds to the increasing evidence about the importance of collaborative practices for ensuring safe medication management among home-dwelling older adults with polypharmacy after hospital discharge.

Our study had some limitations, however. For feasibility reasons, we excluded individuals who could not speak French. This may be considered a limitation given the high migration rates in French-speaking Switzerland. Nevertheless, few individuals were excluded for this specific reason according to the hospital and CHC nurses who supported participant recruitment. Moreover, data collection had to be suspended at the beginning of March 2020 due to the older adults’ and informal caregivers’ vulnerability to the COVID-19 pandemic. Data collection was also suspended among professional caregivers because their skills were in such high demand during this crisis. Given the study’s longitudinal nature, follow-up data from four of the older adults recruited were lost. Adjusting our qualitative methodology by collecting data via an online platform was unfeasible since our research population was mostly unfamiliar with information and communication technologies.

Another limitation noted during interviews was the difficulty keeping older adults and their informal caregivers focused on the theme of medication management rather than on other IADL and ADL. It was also difficult to maintain focus on the most recent care transition and ignore previous hospitalizations. Planning joint interviews between older adults and their professional caregivers was infeasible, although this could have enhanced our data through this direct access to their interactions. Despite our efforts at methodological rigor, we cannot exclude a risk of social desirability bias among study participants. It is thus possible that medication management was, in fact, less effective and collaborative than its portrayal in the interviews.

## Conclusion

Describing the perceived needs of older adults taking several different medications at home, and those of their informal and professional caregivers, for collaborative medication management after hospital discharge revealed several opportunities for improving safety and effectiveness and preventing medication-related harm. Four primary needs were revealed, pointing to two main pathways for improving medication management: firstly, post-discharge medication follow-up by a community healthcare professional skilled in care management, and secondly, empowering older adults and their informal caregivers and sharing decision-making with them regarding medication management.

Further research, applying different research methods, will be required to identify how to meaningfully engage with older adults and their informal and professional caregivers in order to best support safe post-discharge medication management. Moreover, further research involving joint professional interviews or focus groups (bringing together nurses, GPs, and pharmacists) could help to co-design solutions by clarifying each group’s roles, their complementarity, and their understandings of complex medication management situations. Such research would continue to improve our knowledge of and interventions for safer collaborative medication management.

## Electronic supplementary material

Below is the link to the electronic supplementary material.

Supplementary Material 1

Supplementary Material 2

Supplementary Material 3

Supplementary Material 4

## Data Availability

The datasets used and/or analyzed during the current study are available from the corresponding author on reasonable request.

## List of abbreviations

ADL: Basic activities of daily living
CHC: Community healthcare center
GP: General practitioner
IADL: Instrumental activities of daily living
IC: Informal caregiver
ICD-10: 10th revision of the International Statistical Classification of Diseases and Related Health Problems
OA: Older adults
Prof: Healthcare professionals

## References

[1] World Health Organization. Medication without harm. Geneva; 2017.

[2] Tariq RA, Vashisht R, Sinha A, Scherbak Y. Medication dispensing errors and prevention. 2018. 30085607

[3] Masnoon N, Shakib S, Kalisch-Ellett L, Caughey GE (2017). What is polypharmacy? A systematic review of definitions. BMC Geriatr.

[4] Drenth-van Maanen AC, Wilting I, Jansen PA (2020). Prescribing medicines to older people—How to consider the impact of ageing on human organ and body functions. Br J Clin Pharmacol.

[5] Monégat M, Sermet C, Perronnin M, Rococo E. Polypharmacy: definitions, measurement and stakes involved. Review of the literature and measurement tests. 2014.

[6] Al Hamid A, Ghaleb M, Aljadhey H, Aslanpour Z (2014). A systematic review of hospitalization resulting from medicine-related problems in adult patients. Br J Clin Pharmacol.

[7] Nickel CH, Ruedinger JM, Messmer AS, Maile S, Peng A, Bodmer M (2013). Drug - related emergency department visits by elderly patients presenting with non-specific complaints. Scand J Trauma Resusc Emerg Med.

[8] Fallis BA, Dhalla IA, Klemensberg J, Bell CM. Primary medication non-adherence after discharge from a general internal medicine service. PLo e61735 doi101371journalpone0061735. 2013;8. 10.1371/journal.pone.0061735 PMC3642181 23658698

[9] Wong C (2020). Medication-related problems in older people: how to optimise medication management. Hong Kong Medical Journal.

[10] Howland RH (2012). Effective medication management. J PsychoSoc Nurs Ment Health Serv.

[11] Roughead EE, Vitry AI, Caughey GE, Gilbert AL (2011). Multimorbidity, care complexity and prescribing for the elderly. Aging Health.

[12] Gilbert A, Roughead L, McDermott R, Ryan P, Esterman A, Shakib S, et al. Multiple Chronic Health Conditions in Older People: Implications for Health Policy Planning, Practitioners and Patients. University of South Australia; 2013.

[13] Parekh N, Gahagan B, Ward L, Ali K (2019). ‘They must help if the doctor gives them to you’: a qualitative study of the older person’s lived experience of medication-related problems. Age Ageing.

[14] Tomlinson J, Silcock J, Smith H, Karban K, Fylan B (2020). Post-discharge medicines management: the experiences, perceptions and roles of older people and their family carers. Health Expect.

[15] Tomlinson J, Cheong V-L, Fylan B, Silcock J, Smith H, Karban K (2020). Successful care transitions for older people: a systematic review and meta-analysis of the effects of interventions that support medication continuity. Age Ageing.

[16] Holmqvist M, Thor J, Ros A, Johansson L (2021). Evaluation of older persons’ medications: a critical incident technique study exploring healthcare professionals’ experiences and actions. BMC Health Serv Res.

[17] Sandelowski M. Whatever happened to qualitative description? Research in nursing & health. 2000;23(4):334 – 40. 10.1002/1098-240x(200008)23:4<334::aid-nur9>3.0.co;2-g 10940958

[18] Sandelowski M (2010). What’s in a name? Qualitative description revisited. Res Nurs Health.

[19] Braun V, Clarke V (2006). Qualitative Research in Psychology Using thematic analysis in psychology Using thematic analysis in psychology. Qualitative Res Psychol.

[20] Studer H, Boeni F, Hersberger KE, Lampert ML (2021). Pharmaceutical Discharge Management: Implementation in Swiss Hospitals Compared to International Guidelines. Pharmacy.

[21] Vaud Éd. Commission consultative du soutien aux proches aidants | État de Vaud. Site Officiel État de Vaud; 2018.

[22] Bradshaw C, Atkinson S, Doody O (2017). Employing a qualitative description approach in health care research. Global qualitative nursing research.

[23] Roux P, Verloo H, Santiago-Delefosse M, Pereira F (2019). The spatial dimensions of medication management by home-dwelling older adults after hospital discharge. Health Place.

[24] World Health Organization. International Statistical Classification of Diseases and Related Health Problems 10th Revision 2019 [updated 2019. Available from: https://icd.who.int/browse10/2019/en.

[25] Polak L, Green J (2016). Using Joint Interviews to Add Analytic Value. Qual Health Res.

[26] Knight DA, Thompson D, Mathie E, Dickinson A (2013). Seamless care? Just a list would have helped!’Older people and their carer’s experiences of support with medication on discharge home from hospital. Health Expect.

[27] Taylor B, De Vocht H (2011). Interviewing separately or as couples? Considerations of authenticity of method. Qual Health Res.

[28] Braun V, Clarke V (2019). Reflecting on reflexive thematic analysis. Qualitative Res sport Exerc health.

[29] Lincoln YS, Guba EG. Naturalistic inquiry: Sage; 1985.

[30] Mabire C, Büla C, Morin D, Goulet C (2015). Nursing discharge planning for older medical inpatients in Switzerland: a cross-sectional study. Geriatr Nurs.

[31] Siddique SM, Tipton K, Leas B, Greysen SR, Mull NK, Lane-Fall M (2021). Interventions to Reduce Hospital Length of Stay in High-risk Populations: A Systematic Review. JAMA Netw open.

[32] How does the Swiss health system work? [press release]. swissinfo.ch2018.

[33] Lee JI, Cutugno C, Pickering SP, Press MJ, Richardson JE, Unterbrink M (2013). The Patient Care Circle: A Descriptive Framework for Understanding Care Transitions. J Hosp Med.

[34] Giovannini S, Tamburrano A, Sganga F, Serra M, Loreti C, Coraci D (2020). A new model of multidimensional discharge planning: continuity of care for frail and complex inpatients. Eur Rev Med Pharmacol Sci.

[35] Deschodt M, Flamaing J, Haentjens P, Boonen S, Milisen K (2013). Impact of geriatric consultation teams on clinical outcome in acute hospitals: a systematic review and meta-analysis. BMC Med.

[36] Holmqvist M, Thor J, Ros A, Johansson L (2019). Older persons’ experiences regarding evaluation of their medication treatment—An interview study in Sweden. Health Expect.

[37] O’Quin KE, Semalulu T, Orom H (2015). Elder and caregiver solutions to improve medication adherence. Health Educ Res.

[38] Reinhard SC, Levine C, Samis S. Home alone: family caregivers providing complex chronic care. Bmj. 2012:41.

[39] Otobe Y, Suzuki M, Kimura Y, Koyama S, Kojima I, Ichikawa T (2021). Relationship between expression of gratitude by home-based care receivers and caregiver burden among family caregivers. Arch Gerontol Geriatr.

[40] Nah S, Martire LM, Zhaoyang R. Perceived, Gratitude, Role Overload, and Mental Health Among Spousal Caregivers of Older Adults. The Journals of Gerontology: Series B. 2021. 10.1093/geronb/gbab086 PMC8824555 33979437

[41] Cramm JM, Hoeijmakers M, Nieboer AP (2014). Relational coordination between community health nurses and other professionals in delivering care to community-dwelling frail people. J Nurs Adm Manag.

[42] Bindels J, Cox K, Widdershoven G, van Schayck OC, Abma TA (2014). Care for community-dwelling frail older people: a practice nurse perspective. J Clin Nurs.

[43] Takahashi PY, Leppin AL, Hanson GJ, editors. Hospital to Community Transitions for Older Adults: An Update for the Practicing Clinician. Mayo Clinic Proceedings; 2020: Elsevier. 10.1016/j.mayocp.2020.02.001 32736941

[44] Ahmed OI (2016). Disease management, case management, care management, and care coordination: A framework and a brief manual for care programs and staff. Prof case Manage.

[45] Nafradi L, Nakamoto K, Schulz PJ (2017). Is patient empowerment the key to promote adherence? A systematic review of the relationship between self-efficacy, health locus of control and medication adherence. PLoS ONE.

[46] Pulvirenti M, McMillan J, Lawn S (2014). Empowerment, patient centred care and self-management. Health Expect.

[47] Rodakowski J, Rocco PB, Ortiz M, Folb B, Schulz R, Morton SC (2017). Caregiver integration during discharge planning for older adults to reduce resource use: a metaanalysis. J Am Geriatr Soc.

[48] World Health Organization. Continuity and coordination of care: a practice brief to support implementation of the WHO Framework on integrated people-centred health services. 2018.

