# Supplementary figures and images for: Collaborative medication management for older adults after hospital discharge: a qualitative descriptive study
Source: BMC Nurs. 2022 Oct 24;21:284. doi: 10.1186/s12912-022-01061-3 (PMC9590396; doi:10.1186/s12912-022-01061-3)

**Supplementary File 1**

*Description of the Enrolment Process*


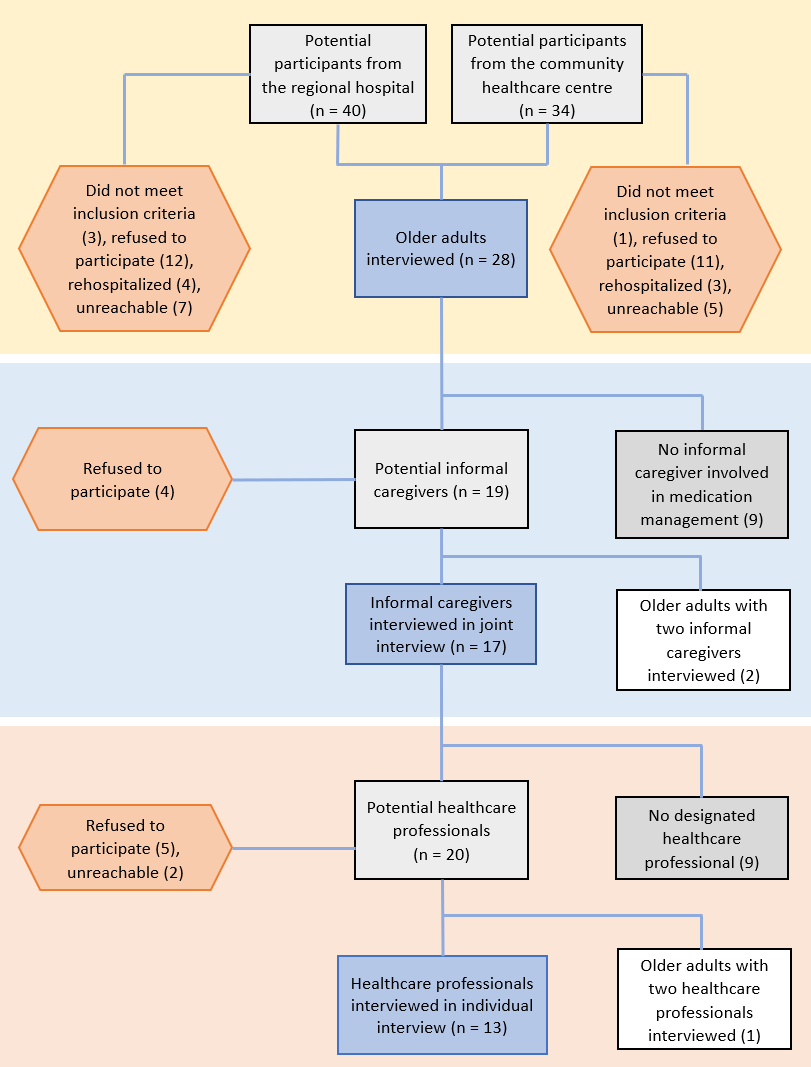

Supplement: Supplementary file 1 — Supplementary Material 1 [file 12912_2022_1061_MOESM1_ESM.docx]
