# Supplementary material for: Collaborative medication management for older adults after hospital discharge: a qualitative descriptive study
Source: BMC Nurs. 2022 Oct 24;21:284. doi: 10.1186/s12912-022-01061-3 (PMC9590396; doi:10.1186/s12912-022-01061-3)
Supplement: Supplementary file 2 — Supplementary Material 2 [file 12912_2022_1061_MOESM2_ESM.docx]

**Supplementary File 3**

*Older Adult Participants’ Characteristics*

| Older adult ID | Age | Sex | Marital status | Living with | Hospital length of stay (days) | Number of  ICD-10 diagnoses | Number of medications | Principal ICD-10 diagnosis for hospitalization |
| --- | --- | --- | --- | --- | --- | --- | --- | --- |
| OA01 | 92 | F | widow | alone | 6 | 7 | 12 | M80.88 Other osteoporosis with current pathological fracture, vertebra(e) |
| OA02 | 66 | M | married | spouse | 112 | 27 | 21 | B25.80 Cytomegaloviral disease of the digestive system |
| OA03 | 73 | F | married | spouse | 39 | 12 | 8 | C16.3 Malignant tumor: pyloric antrum |
| OA04 | 82 | F | divorced | alone | 20 | 25 | 14 | S70.0 Contusion of hip |
| OA05 | 71 | F | widow | alone | 60 | 12 | 6 | K92.1 Melena |
| OA06 | 86 | M | married | spouse | 14 | 10 | 9 | J18.0 Bronchopneumonia, unspecified |
| OA07 | 94 | F | widow | alone | 12 | 10 | 8 | I50.01 Right ventricular failure (secondary to left heart failure) |
| OA08 | 73 | F | widow | children | 12 | 3 | 10 | N30.0 Acute cystitis |
| OA09 | 83 | F | widow | alone | 37 | 14 | 8 | T84.04 Mechanical complication of internal joint prosthesis: hip joint |
| OA10 | 85 | M | widower | alone | 18 | 18 | 7 | M80.98 Unspecified osteoporosis with pathological fracture |
| OA11 | 82 | M | married | spouse | 41 | 18 | 13 | D64.8 Other specified anemias |
| OA12 | 82 | M | widower | children | 14 | 17 | 8 | I50.01 Right ventricular failure (secondary to left heart failure) |
| OA13 | 75 | M | widower | alone | 14 | 9 | 7 | I63.0 Cerebral infarction due to thrombosis of precerebral arteries |
| OA14 | 88 | F | widow | alone | 17 | 10 | 7 | S22.4 Multiple fractures of ribs |
| OA15 | 85 | M | married | spouse | 34 | 16 | 13 | I63.4 Cerebral infarction due to embolism of cerebral arteries |
| OA17 | 87 | M | married | spouse/  children | 47 | 22 | 11 | K80.30 Calculus of bile duct with cholangitis |
| OA18 | 86 | M | married | spouse | 13 | 5 | 5 | I63.8 Other cerebral infarction |
| OA19 | 84 | M | married | spouse | 5 | 9 | 6 | G45.0 Vertebrobasilar artery syndrome |
| OA20 | 69 | M | divorced | alone | 13 | 14 | 13 | I74.3 Embolism and thrombosis of arteries of lower extremities |
| OA21 | 75 | F | married | spouse | 13 | 16 | 6 | R26.8 Other and unspecified abnormalities of gait and mobility |
| OA22 | 83 | F | widow | alone | 8 | 5 | 7 | J13 Pneumonia due to Streptococcus pneumoniae |
| OA23 | 87 | F | widow | alone | 5 | 8 | 11 | A08.1 Acute gastroenteropathy due to Norwalk agent |
| OA24 | 74 | M | married | spouse | 8 | 13 | 9 | J18.1 Lobar pneumonia, unspecified |
| OA25 | 88 | M | married | spouse | 12 | 18 | 7 | I50.01 Left ventricular failure |
| OA26 | 76 | M | married | spouse | 1 | 4 | 4 | C22.0 Liver cell carcinoma |
| OA27 | 77 | M | married | spouse | 12 | 11 | 8 | C20 Malignant neoplasm of rectum |
| OA28 | 89 | M | widower | alone | 14 | 9 | 14 | K56.5 Intestinal adhesions [bands] with obstruction (post-infection) |
| OA29 | 81 | M | married | spouse | 4 | 9 | 8 | K80.20 Calculus of gallbladder without cholecystitis without obstruction |
