# Supplementary material for: Collaborative medication management for older adults after hospital discharge: a qualitative descriptive study
Source: BMC Nurs. 2022 Oct 24;21:284. doi: 10.1186/s12912-022-01061-3 (PMC9590396; doi:10.1186/s12912-022-01061-3)
Supplement: Supplementary file 3 — Supplementary Material 3 [file 12912_2022_1061_MOESM3_ESM.docx]

**Supplementary File 4**

*Informal and professional caregivers’ characteristics*

| Older adult ID | Informal caregiver ID | Age | Sex | Relationship | Healthcare Professional ID | Profession | Age | Sex | |
| --- | --- | --- | --- | --- | --- | --- | --- | --- | --- |
| OA01 | IC01a  IC01b | 59  67 | F  F | Daughter  Daughter | Prof01a  Prof01b | Pharmacist Pharmacy assistant | 40  28 | | F  F |
| OA02 | IC02 | 58 | F | Wife | No designated healthcare professional | | | | |
| OA03 | IC03 | 71 | M | Husband | Prof03 | Nurse | 30 | | F |
| OA04 | IC04 | 54 | F | Daughter | Prof04 | Nurse | 43 | | F |
| OA05 | Not involved in medication management | | | | No designated healthcare professional | | | | |
| OA06 | Refused to participate | | | | No designated healthcare professional | | | | |
| OA07 | IC07 | 52 | F | Daughter-in-law | Prof07 | Nurse | 31 | | M |
| OA08 | IC08 | 55 | F | Daughter | Prof08 | Pharmacist | 56 | | F |
| OA09 | Not involved in medication management | | | | No designated healthcare professional | | | | |
| OA10 | Not involved in medication management | | | | Refused to participate | | | | |
| OA11 | IC11 | 57 | F | Wife | Refused to participate | | | | |
| OA12 | IC12 | 48 | F | Daughter | No designated healthcare professional | | | | |
| OA13 | Not involved in medication management | | | | Refused to participate | | | | |
| OA14 | Refused to participate | | | | No designated healthcare professional | | | | |
| OA15 | IC15 | 84 | F | Wife | Prof15 | Nurse | 58 | | F |
| OA17 | IC17a  IC17b | 85  52 | F  F | Wife  Daughter | Prof17 | GP | 54 | | F |
| OA18 | IC18 | 86 | F | Wife | Prof18 | Pharmacist | 54 | | M |
| OA19 | Refused to participate | | | | No designated healthcare professional | | | | |
| OA20 | Refused to participate | | | | Prof20 | Nurse | 46 | | F |
| OA21 | IC21 | 80 | M | Husband | Prof21 | GP | 39 | | M |
| OA22 | Not involved in medication management | | | | Refused to participate | | | | |
| OA23 | Not involved in medication management | | | | No designated healthcare professional | | | | |
| OA24 | Not involved in medication management | | | | Refused to participate | | | | |
| OA25 | IC25 | 76 | F | Wife | Unreachable | | | | |
| OA26 | IC26 | 75 | F | Wife | No designated healthcare professional | | | | |
| OA27 | IC27 | 80 | F | Wife | Unreachable | | | | |
| OA28 | Not involved in medication management | | | | Prof28 | GP | - | | F |
| OA29 | Not involved in medication management | | | | Prof29 | Oncologist | 47 | | F |
