# Supplementary material for: Collaborative medication management for older adults after hospital discharge: a qualitative descriptive study
Source: BMC Nurs. 2022 Oct 24;21:284. doi: 10.1186/s12912-022-01061-3 (PMC9590396; doi:10.1186/s12912-022-01061-3)
Supplement: Supplementary file 4 — Supplementary Material 4 [file 12912_2022_1061_MOESM4_ESM.docx]

**Supplementary File 2**

*Interview guides*

The interview guides were inspired by our literature review and tested in a preliminary study [1]. The original French versions were approved by the Human Research Ethics Committee of the Canton of Vaud on 5 July 2017 (2017-01025) and on 1 February 2019 (2018-02196), as well as by the institutional review board of our study’s field partners. The guides have been translated into English for publication.

**Interview 1: Older adult**

| Topics | Items |
| --- | --- |
| 1) Presentation | Presentation of the study  Presentation of the interview’s objectives  Details of the ethics measures taken |
| 2) Experience of hospitalization and hospital discharge | General experience of the hospital stay  Experience with medication received in hospital:   - Changes to usual treatment in hospital - Information received in hospital about medication changes - People involved - Tools received in hospital to help manage medication at home |
| 3) Experience of the return home | General experience and process of return home  Experience with medication since returning home:   - Management of medicines (and any changes made in hospital) since return home - People involved in medication management at home - Perceptions/experiences of taking several medications per day - Methods put in place to avoid forgetting to take medication, keep to the right schedule, and avoid taking the wrong medication - Taking other health or wellness products |
| 5) Socio-demographic data |  |
| 6) End of the interview | Reminder of the ethical requirements for using the data collected in the interview |

**Interview 2 (follow up interview): Older adult**

| Topics | Items |
| --- | --- |
| 1) Presentation | Presentation of the interview’s objectives  Reminder of the details of the ethics measures taken |
| 2) Daily medication management | Description of daily medication management:   - Medication management locations (taking, storing) - Schedules - Routines |
| 4) Support at home for medication management | People involved in the day-to-day management of medicines:   - Who (people involved) - Frequency of assistance - Type of assistance |
| 3) Experiences with medication | Medication habits and changes:   - Time of onset of medication (before, during and after hospitalization)   Knowledge about medications:   - Indication for each medication - Effects of each medication - Possible precautions - Most important medication   Satisfaction with information received about each medication  Wish to ask questions about a particular medication |
| 4) End of the interview | Reminder of the ethical requirements for using the data collected in the interview |

**Interview 3: Joint interview with the older adult and their informal caregiver**

| Topics | Items |
| --- | --- |
| 1) Presentation | Presentation of the study  Presentation of the interview’s objectives  Details of the ethics measures taken |
| 2) Older adult–informal caregiver relationship | The relationship between them  Assistance provided in ADL and IADL |
| 3) Experience of the return home | Process of hospital discharge and return home:   - Experience with medication changes - Information received - Experience with medication since returning home - Support for medication management |
| 4) Involvement in medication management | Activities where the informal caregiver is involved in medication management  How it happens  Example |
| 5) Sociodemographic data |  |
| 6) End of the interview | Reminder of the ethical requirements for using the data collected in the interview |

**Interview 4: Professional caregiver involved in medication management**

| Topics | Items |
| --- | --- |
| 1) Presentation | Presentation of the study  Presentation of the interview’s objectives  Details of the ethics measures taken |
| 2) Experience with the older adult in relation to medication management | Since when has Mr./Mrs. X been followed  Type of intervention(s) for medication management (prescription, preparation, administration, monitoring, etc.)  Frequency of intervention  Process of hospital discharge and the return home:   - Information received before the first visit after hospitalization - Possible changes in usual medication   Progress of the medication adjustment  Any difficulties encountered with Mr./Mrs. X regarding medication management |
| 3) Sociodemographic and professional data |  |
| 4) End of the interview | Reminder of the ethical requirements for using the data collected in the interview |

1. Roux P, Verloo H, Santiago-Delefosse M, Pereira F. The spatial dimensions of medication management by home-dwelling older adults after hospital discharge. Health Place. 2019;60:102230.
